# The effect of the ^13^C abundance of soil microbial DNA on identifying labelled fractions after ultracentrifugation

**DOI:** 10.1007/s00253-024-13151-0

**Published:** 2024-05-03

**Authors:** Juan Wang, Huaiying Yao, Xian Zhang

**Affiliations:** 1 Key Laboratory of Urban Environment and Health, Institute of Urban Environment, Chinese Academy of Sciences, Xiamen, China; 2 Zhejiang Key Laboratory of Urban Environmental Processes and Pollution Control, CAS Haixi Industrial Technology Innovation Center in Beilun, Ningbo, China; 3 Research Center for Environmental Ecology and Engineering, School of Environmental Ecology and Biological Engineering, Wuhan Institute of Technology, Wuhan, China

**Keywords:** DNA-SIP, Method, 16S rRNA gene, 18S rRNA gene, Soil sample

## Abstract

**Abstract:**

DNA-based stable isotope probing (DNA-SIP) technology has been widely employed to trace microbes assimilating target substrates. However, the fractions with labelled universal genes are sometimes difficult to distinguish when detected by quantitative real-time PCR. In this experiment, three paddy soils (AQ, CZ, and NB) were amended with 0.1% glucose containing ^13^C at six levels, and DNA was then extracted after a 7-day incubation and subjected to isopycnic gradient centrifugation. The results showed that the amount of labelled DNA was notably related to the ^13^C-glucose percentage, while the separation spans of 18S rRNA and 16S rRNA genes between labelled and unlabelled treatments became notably clearer when the δ^13^C values of the total DNA were 90.9, 61.6, and 38.9‰ and 256.2, 104.5 and 126.1‰ in the AQ, CZ, and NB soils, respectively. Moreover, fractionated DNA was also labelled by determining the δ^13^C values while adding only 5 atom% ^13^C-glucose to the soil. The results suggest that the optimal labelling fractions were not always those fractions with the maximal gene abundance, and detecting the δ^13^C values of the total and fractionated DNA was beneficial in estimating the results of DNA-SIP.

**Key points:**

*• Appropriate* ^13^ *C-DNA amount was needed for DNA-SIP.*

*• Detecting the* ^13^ *C ratio of fractionated DNA directly was an assistant method for identifying the labelled fractions.*

*• Fractions with the maximal 18S or 16S rRNA gene abundance always were not labelled.*

## Introduction

The DNA-based stable isotope probing (DNA-SIP) technique was developed to examine specific microbes while assimilating isotopically labelled substrates in complicated ecological environmental samples, such as soils and sediments (Bao et al. 2022; Li et al. 2019, 2018; Long et al 2018; Radajewski et al. 2000). After labelling, the main procedure of this method is as follows: (I) extracting microbial DNA, (II) mixing the DNA with a CsCl solution and adjusting the buoyant density of the mixture, (III) conducting isopycnic density gradient centrifugation, (IV) separating the fractions based on the buoyant density, (V) identifying the fraction containing the labelled gene, and (VI) analyzing the target gene. Therefore, the key factor in executing DNA-SIP is separating the labelled fractions and identifying the labelled gene.

After ultracentrifugation, the earliest report introduced a method relying on ethidium bromide (EB) staining and a second ultracentrifugation step to identify and purify the ^13^C-DNA fraction (Radajewski et al. 2000). As this approach was based on a large amount of DNA, which was always difficult to obtain from soil, Lueders et al. (2004) developed a relatively sensitive method to estimate the gene abundance in isopycnic gradient fractions by quantification of the 16S rRNA genes in gradient fractions via real-time PCR. Subsequently, qPCR has been widely implemented to identify fractions with labelled genes. Ideally, a high gene abundance tends to move towards the high buoyant density of labelled samples compared with unlabelled treatments, and the fraction with a relatively high gene abundance among the labelled samples would be regarded as containing the microbes responsible for assimilating the labelled materials (Bao et al. 2022; Yang et al. 2022; Beulig et al. 2015; Hu and Lu 2015).

The target gene studied in previous studies could be classified into two genres: one is functional genes, such as particulate methane monooxygenase (pmoA) or ammonia monooxygenase (amoA) genes, and the other is universal genes, such as bacterial 16S rRNA or fungal 18S rRNA genes. For the functional genes, the span of the qPCR results between labelled and unlabelled treatments was large enough to easily predict the labelled fractions (Dillon et al. 2022; Liu et al. 2023; Sun et al. 2022; Li et al. 2022). For the universal genes, the span of the qPCR results was not conclusive in confirming the labelled fractions with a relatively high gene abundance due to the low rate of substrate turnover in certain studies (Li et al. 2022; Wang et al. 2019; Xu et al. 2019). Furthermore, with the development of the high-throughput sequencing (HTS) technique, certain studies have defined heavy and light fractions purely by the density (Da Costa et al. 2018; Gkarmiri et al. 2017; Zhang et al. 2015), and some studies have relied on the carrier (pure culture) to identify the labelled fractions (Liu et al. 2016; Sietio et al. 2018; Sun et al. 2018), while other studies have sequenced fractions directly (Hannula et al. 2017; Pepe-Ranney et al. 2016). Considering these choices, either deciding on the labelled fractions arbitrarily or increasing the subsequent analysis intensity to most fractions, it is still necessary to identify the fractions with labelled universal genes as precisely as possible.

In some approaches, the apparent shift in DNA concentration to a heavy buoyant density was determined, and labelled fractions were readily evaluated when soil was labelled with exopolysaccharides (Wang et al. 2015), residues (Rime et al. 2016), ^13^CO_2_ (Beulig et al. 2015), or root exudates (Mao et al. 2014), although this method is less representative and sensitive than qPCR. The ultracentrifugation conditions and gene-labelled extent were the main factor in the separation result between labelled and unlabeled treatments. While the centrifugation time, speed, and initial buoyant density were identified suitable in most of the studies (Wang et al. 2020), the required amount of substrate for the detection of the labelled fraction via qPCR and the required extent of the shift in fraction (or span) to confirm the labelled fraction are still unclear. Therefore, six levels of ^13^C-labelled glucose were amended to three paddy soils, and isopycnic density gradient centrifugation and qPCR of the 16S and 18S rRNA genes were subsequently conducted in this study. Moreover, a direct method of determining the δ^13^C values of DNA before and after ultracentrifugation was also employed to help distinguish the labelled fractions (Haichar et al. 2012; Hou et al. 2018). This experiment should provide helpful insights for studies employing the DNA-SIP technique to avoid superfluous operation and analysis.

## Materials and methods

### Soil sample collection

The three paddy soil samples were collected in winter from Anqing (AQ, 116° 17′ 49″ E, 30° 26′ 03″ N), Anhui Province; Changzhou (CZ, 119° 51′ 54″ E, 31° 55′ 16″ N), Jiangsu Province; and Ningbo (NB, 121° 51′ 56″ E, 29° 45′ 10″ N), Zhejiang Province. After field sampling, the soils were air-dried, straw and root debris were removed, and the soils were sieved (2-mm mesh). The basic and chemical properties of the three soils are listed in Table 1.

**Table 1:** Chemical properties of the three soils

Soil	pH	Organic matter (g kg^−1^)	Total N content (g kg^−1^)	Total P content (g kg^−1^)	Total K content (g kg^−1^)
AQ	6.21	18.8	0.81	1.74	15.87
CZ	6.60	21.9	1.47	0.66	9.06
NB	7.91	28.0	1.2	0.95	21.52

### Soil microcosms

The soil was adjusted to a water-holding capacity (WHC) of 50% and preincubated at 25 °C for 7 days. Microcosms were established in 120-mL serum bottles with the equivalent of 20 g dried soil and 0.8 mL 0.025 g mL^−1^ glucose solution, which contained 0, 0.04, 0.08, 0.24, 0.4, or 0.8 mL of a 0.025 g mL^−1 13^C-glucose solution (99% atom ^13^C) and 0.8, 0.76, 0.72, 0.56, 0.4, or 0 mL of a 0.025 g mL^−1 12^C-glucose solution (1.07% atom ^13^C). For every soil, there were six treatments amended with 0.1% glucose, and the proportions of ^13^C-glucose were approximately 1.1, 6.0, 10.9, 30.5, 50.0, and 99.9% and denoted by 0, 5, 10, 30, 50, and 100, respectively. Every treatment was performed in triplicate. The serum bottles were sealed with a butyl stopper, and the soil was then incubated at 25 °C. The bottles were opened every 2 days for ventilation. After a 7-day incubation period, the soil was removed and stored at − 80 °C for subsequent analysis.

### SIP gradient fractionation

The total DNA of the soil was extracted with a FastDNA spin kit for soil (MP Biomedicals, Cleveland, OH, USA) according to the manufacturer’s protocol. The DNA concentration was measured with a NanoDrop ND-2000 UV–visible light spectrophotometer (NanoDrop Technologies, Wilmington, DE, USA).

Approximately 3 μg DNA was mixed with 4.4 mL CsCl stock solution (1.88 g mL^−1^ at 20 °C) and 1.1 mL gradient buffer (GB, 0.1 M Tris–HCl, 0.1 M KCl, and 1 mM EDTA, with a pH of 8.0). The mixture was adjusted with a CsCl or GB solution until the buoyant density reached 1.71 g mL^−1^, which was determined with a DR-A1 digital Abbe refractometer (Atago Co., Ltd., Tokyo, Japan). Then, the mixture was transferred to 5.1-mL centrifuge tubes (Beckman, cat. no. 342412) and centrifuged at 20 °C for 48 h in a Beckman Coulter Optima XPN-80 ultracentrifuge on an NVT 65.2 rotor (Beckman Coulter, USA) at 45,000 rpm (~ 184,000 g). Centrifugation was stopped with no braking, and sterile water was then injected from the top of the ultracentrifuge tube to replace the gradient medium. The injection speed of the syringe was controlled by an LSP01-2A single syringe pump (LongerPump®, Baoding, Hebei, China) at a precise flow rate of 290 μL min^−1^. The outflow solution was collected with a BSZ-100 fraction collector (Shanghai Jiapeng Technology Co., Ltd., China), and a total of 16 gradient fractions were generated. After determining the buoyant density, the fractionated DNA was twice purified with 500 μL 70% ethanol after 550 μL polyethylene glycol (PEG) 6000 precipitation for 2 h. Finally, the DNA was air-dried and dissolved in 30 μL sterilized ultrapure water.

### Quantification of the 16S rRNA and 18S rRNA genes

The abundance of the bacterial 16S rRNA and fungal 18S rRNA genes in the fractionated DNA was determined by quantitative real-time PCR (qPCR) carried out on a LightCycler® 480 II (Roche, Switzerland). For the qPCR, 2 μL of fractionated DNA solution was mixed with 10 μL of 2 × GoTaq® qPCR Master Mix (Promega (Beijing) Biotech Co., Ltd., China), 0.4 μL of each primer (10 μM), and 7.2 μL of sterile water. The primers for the 16S rRNA and 18S rRNA genes were 515F (GTGCCAGCMGCCGCGGTAA) and 907R (CCGTCAATTCMTTTRAGTTT) (Haichar et al. 2012; Hou et al. 2018) and Fung5r (GTAAAAGTCCTGGTTCCCC) and EF4f (GGAAGGGRTGTATTTATTAG) (Haichar et al. 2012; Hou et al. 2018), respectively. All reactions started with initial denaturation at 95 °C for 5 min, followed by amplification and determination of the melting curve. The amplification process for the 16S rRNA and 18S rRNA genes was conducted as follows: 40 cycles at 95 °C for 10 s, maintained at 53 °C for 45 s, maintained at 72 °C for 45 s, and maintained at 84 °C for 15 s to collect signals and 45 cycles at 95 °C for 15 s, maintained at 55 °C for 40 s, maintained at 72 °C for 45 s, and maintained at 84 °C for 15 s to collect signals. The melting curve was obtained by heating from 65 to 97 °C at a rate of 0.11 °C/s. Tenfold serial dilutions of known copy numbers of the corresponding plasmid DNA were used to produce a standard curve. The false positives of the negative (no template) control for the 16S rRNA gene were approximately one percentage of the lowest abundance of the fraction, and the negative control for the 18S rRNA gene showed a negative result.

### Isotope ratio determination

The δ^13^C values of DNA samples before centrifugation and the fractionated DNA of at least one sample of each triplicate were measured through isotope ratio mass spectrometry (IRMS; Delta V advantage) in conjunction with a Flash 2000 HT elemental analyzer (EA) connected to a ConFlo IV (all Thermo Fisher Scientific, USA). Two microliters of the total DNA or 5 μL of the fractionated DNA was placed in a 48-μL tin capsule and dried for 3 h at 55 °C. Then, the tin capsules were placed in an autosampler linked with an EA-IRMS (Thermo Fisher Scientific, USA). Urea was the control sample, and it was determined in triplicate before the DNA samples and after every ten samples, and the δ^13^C value of urea was − 40 ± 0.32‰. The corrected CO_2_ was adopted as a reference, and the δ^13^C content (‰) was determined using the following equation:

$${\updelta }^{13}\mathrm{C }(\mathrm{\permille })=\left[\left({\text{R}}_\text{sample}-{\text{R}}_{\mathrm{standard}}\right)/{\text{R}}_{\mathrm{standard}}\right]\times1000$$

where *R* = ^13^C/^12^C. *R*_standard_ is the Vienna Pee Dee Belemnite standard (V-PDB = 0.0111802).

### Statistical analyses

A linear regression between the δ^13^C values of the total DNA and the amended ^13^C-glucose level in the samples was conducted in Microsoft Excel 2013, and the correlation between the δ^13^C values of the fractionated DNA and qPCR results of the genes was calculated with IBM SPSS Statistics 19.

## Results

**Fig. 1:**
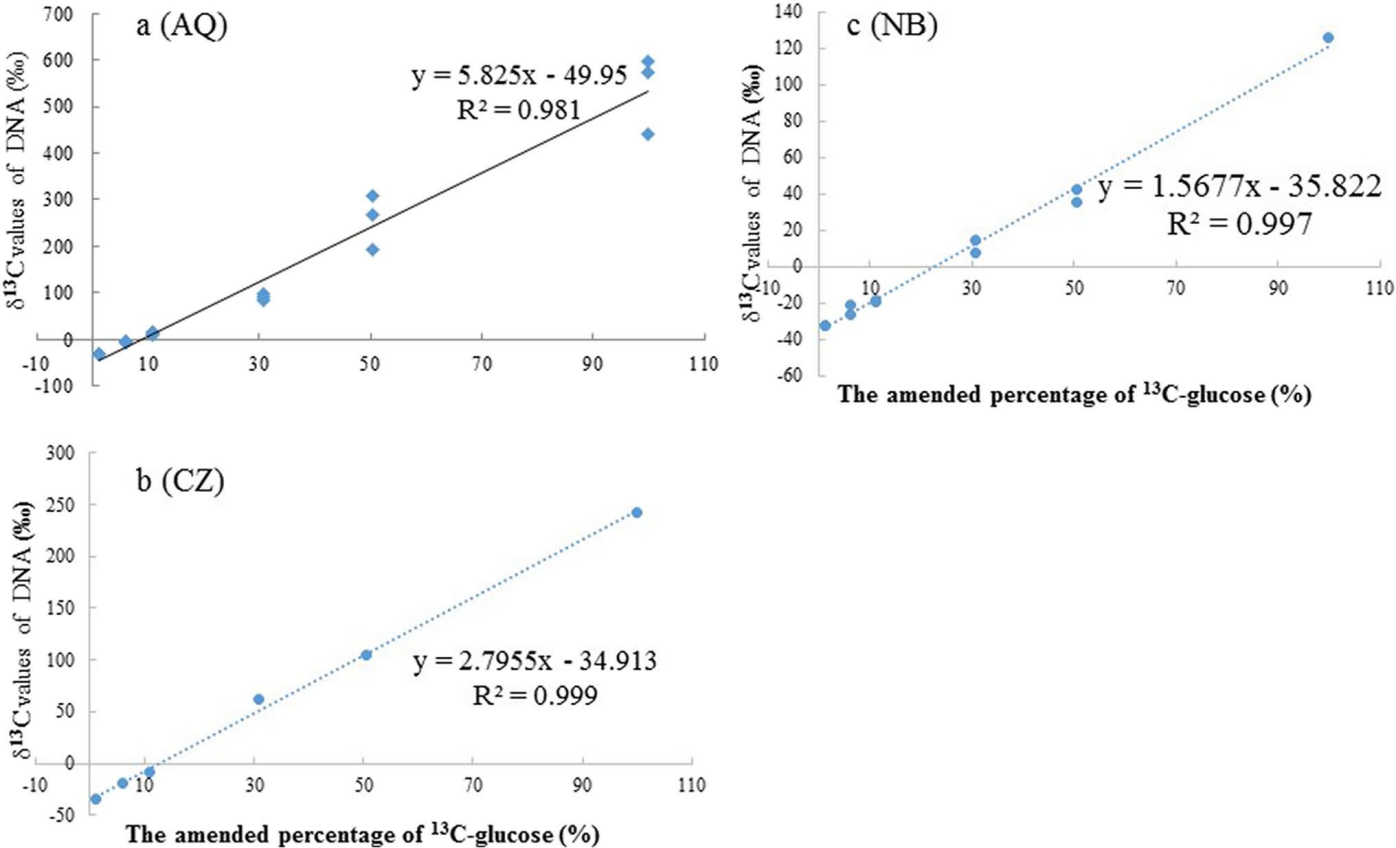
The relationship between the δ^13^C values of DNA and the percent of ^13^C-glucose amendment in AQ (**a**), CZ (**b**), and NB (**c**) soils

The linear regression results revealed that a significant relationship in terms of the ^13^C abundance occurred between the glucose amendment level and the microbial DNA extracted from the soil after 1 week of incubation (Fig. 1). However, the DNA δ^13^C values were different among the different soils at the same ^13^C glucose amendment level. The highest ^13^C degree of labelling occurred in the AQ paddy soil, where the atom% ^13^C level of the microbial DNA increased from 1.08 to 1.72% after a week incubation with 99% ^13^C glucose (Fig. 1a). Otherwise, the labelling DNA amount of CZ was higher than that of the NB paddy soil, and both paddy soils had a labelling amount that was lower than approximately half of the labelling amount determined in the AQ paddy soil.

**Fig. 2:**
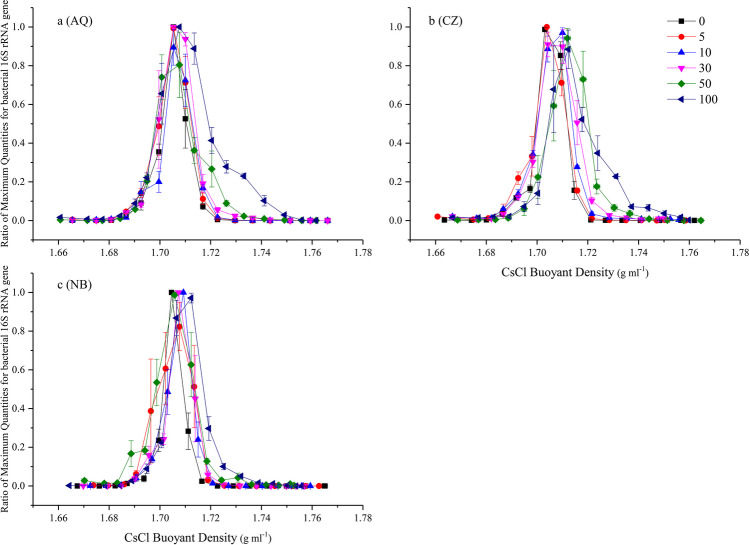
The quantitative distribution of the bacterial 16S rRNA genes across the entire buoyant density gradient of the DNA fractions from soil amended with 0.1% glucose for 7 days with six levels of.^13^C-glucose in AQ (**a**), CZ (**b**), and NB (**c**) soils. Bars represent the standard deviation of the mean (*n* = 3)

**Fig. 3:**
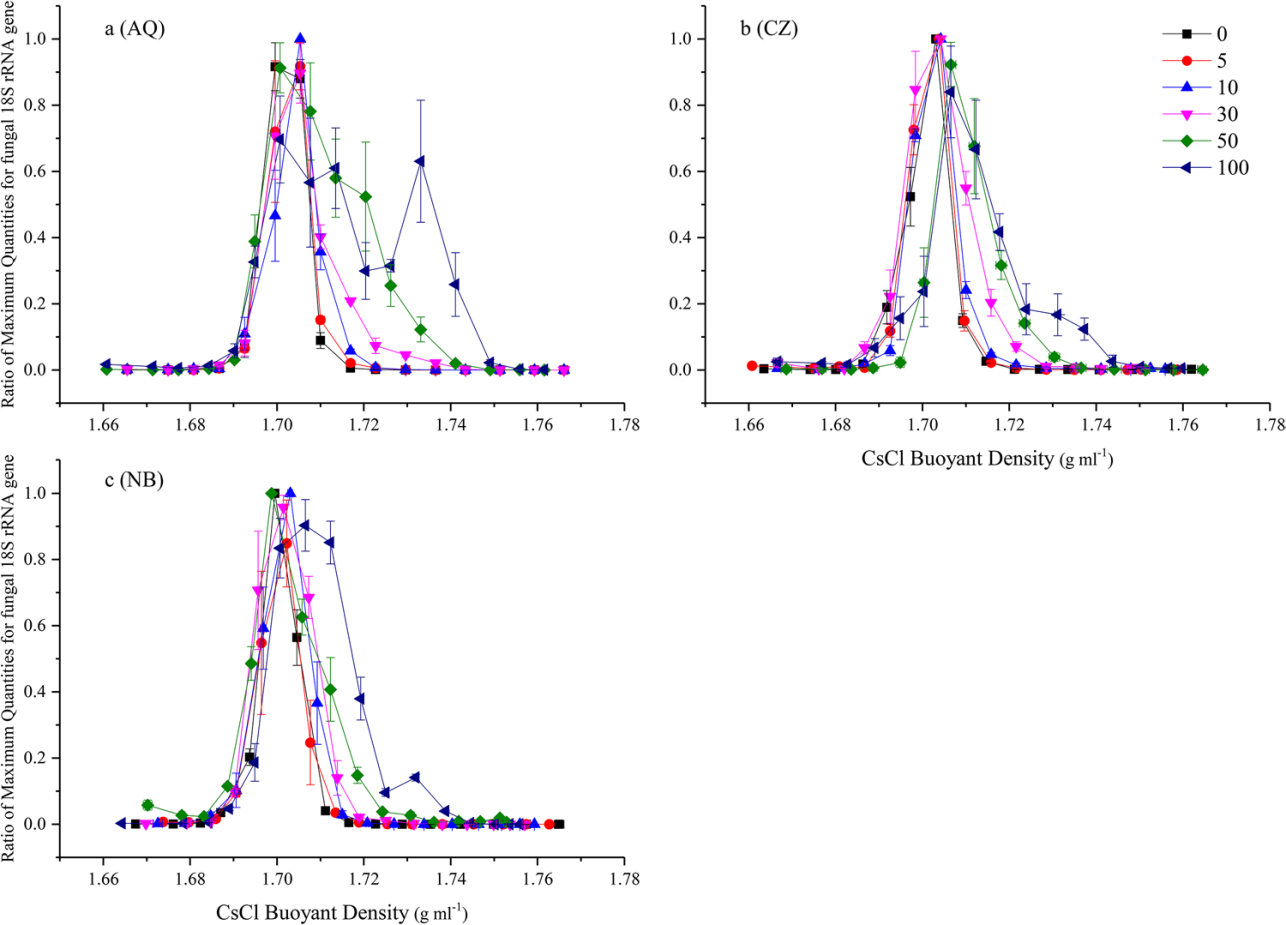
The quantitative distribution of the fungal 18S rRNA genes across the entire buoyant density gradient of the DNA fractions from soil amended with 0.1% glucose for 7 days with six levels of.^13^C-glucose in AQ (**a**), CZ (**b**), and NB (**c**) soils. Bars represent the standard deviation of the mean (*n* = 3)

After ultracentrifugation, the maximum abundances of the 16S rRNA and 18S rRNA genes were concentrated at ~ 1.705 and 1.700 g mL^−1^in the AQ soil, respectively (Figs. 2a and 3a, respectively), and they were both ~ 1.703 g mL^−1^ in the CZ soil (Figs. 2b and 3b, respectively) and 1.704 g mL^−1^ in the NB soil (Figs. 2c and 3c, respectively) for the unlabelled samples. As expected, the most clearly labelled fraction was not the fraction with the maximum gene abundance in most of the treatments. When pure ^13^C-labelled glucose was added, the largest shift in the heavy-fraction 16S rRNA genes was almost 0.036 g mL^−1^ in the AQ soil (Fig. 2a), 0.033 g mL^−1^ in the CZ soil (Fig. 2b), and 0.020 g mL^−1^ in the NB soil (Fig. 2c), while it was 0.041, 0.037, and 0.032 g mL^−1^ for the 18S rRNA genes in the AQ, CZ, and NB soils, respectively (Fig. 3).

Clearly, a higher ^13^C-glucose concentration corresponded to a larger span of the 16S rRNA and 18S rRNA gene relative abundance between the labelled and unlabelled treatments. This finding implied that the labelled fraction was difficult to separate from the unlabelled sample when the content of added ^13^C-labelled glucose was only 5%; at the same time, in the AQ, CZ, and NB soils, the DNA δ^13^C values were − 4.8, − 19.2, and − 21.6‰, respectively, while the corresponding natural DNA δ^13^C values were − 30.7, − 33.6, and − 32.8‰, respectively. For the 16S rRNA gene, when the content of added ^13^C-glucose in AQ and CZ reached 50% and pure ^13^C-labelled glucose was amended in the NB soil, it was relatively easy to distinguish the labelled fractions, and the corresponding DNA δ^13^C values were 256.2, 104.5, and 126.1‰ in the AQ, CZ, and NB soils, respectively. For the 18S rRNA gene, when the content of added ^13^C-glucose in AQ and CZ reached 30%, and 50% ^13^C-labelled glucose was used in the NB soil, the labelled fractions could be visually distinguished, and the corresponding DNA δ^13^C values were 90.9 and 61.6 and 38.9‰ in the AQ, CZ, and NB soils, respectively.

**Fig. 4:**
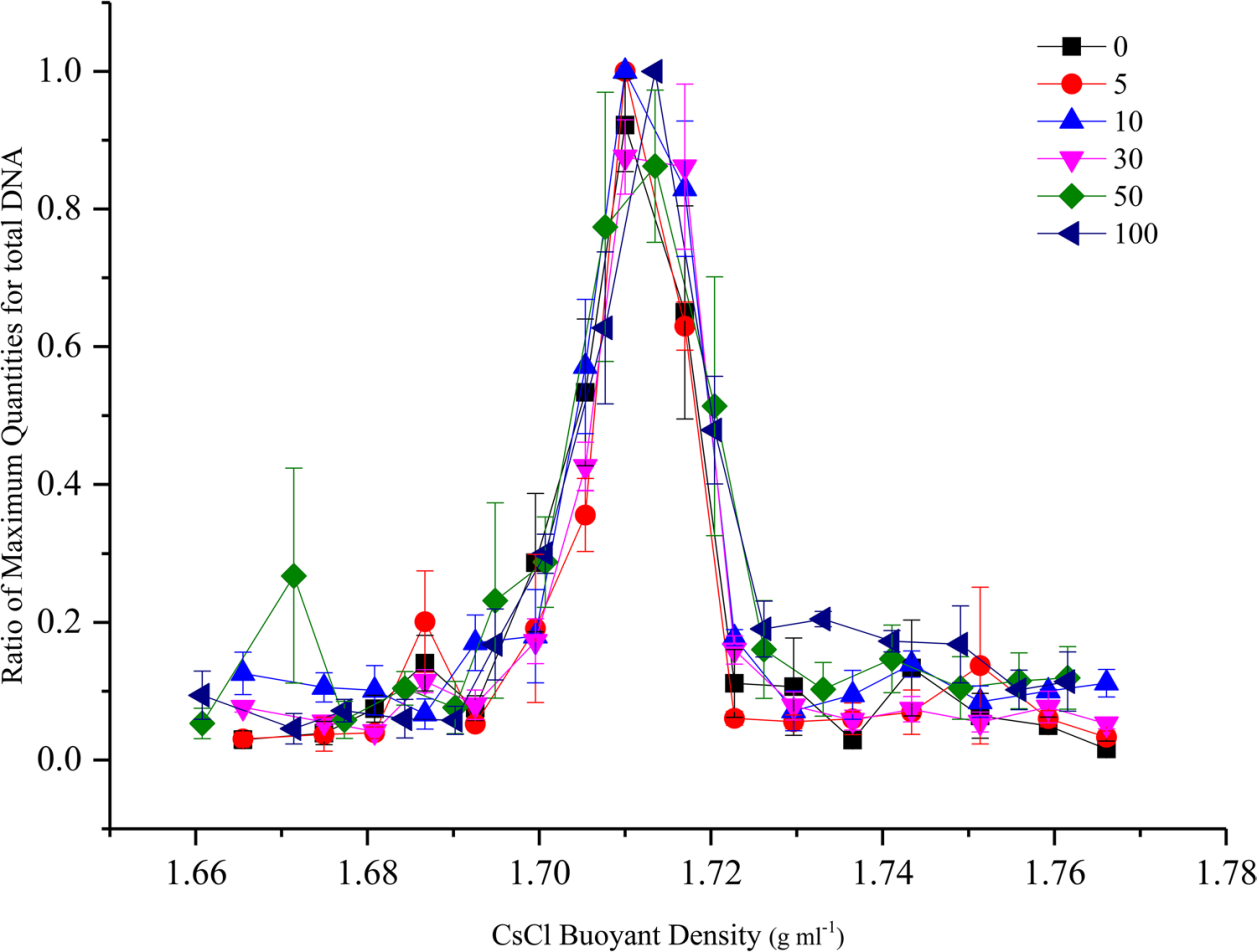
The distribution of the total DNA concentration across the entire buoyant density gradient of the DNA fractions from AQ soil amended with six levels of.^13^C-glucose. Bars represent the standard deviation of the mean (*n* = 3)

Considering that some research has identified ^13^C-labelled DNA by directly detecting the DNA concentration of each fraction, the DNA concentration of each fraction was also measured for the AQ soil. The results demonstrated that the labelled fractions could be separated only in the treatments with 50% and 100% ^13^C-glucose (Fig. 4). Practically, assessing the location of the labelled fractions based on the DNA concentration compared poorly with qPCR results, because the span between the ^12^C and ^13^C treatments was not as distinct as that in the qPCR results, even with 100% labelling. To evaluate the labelling status, the δ^13^C value of each fraction after ultracentrifugation was directly measured.

**Fig. 5:**
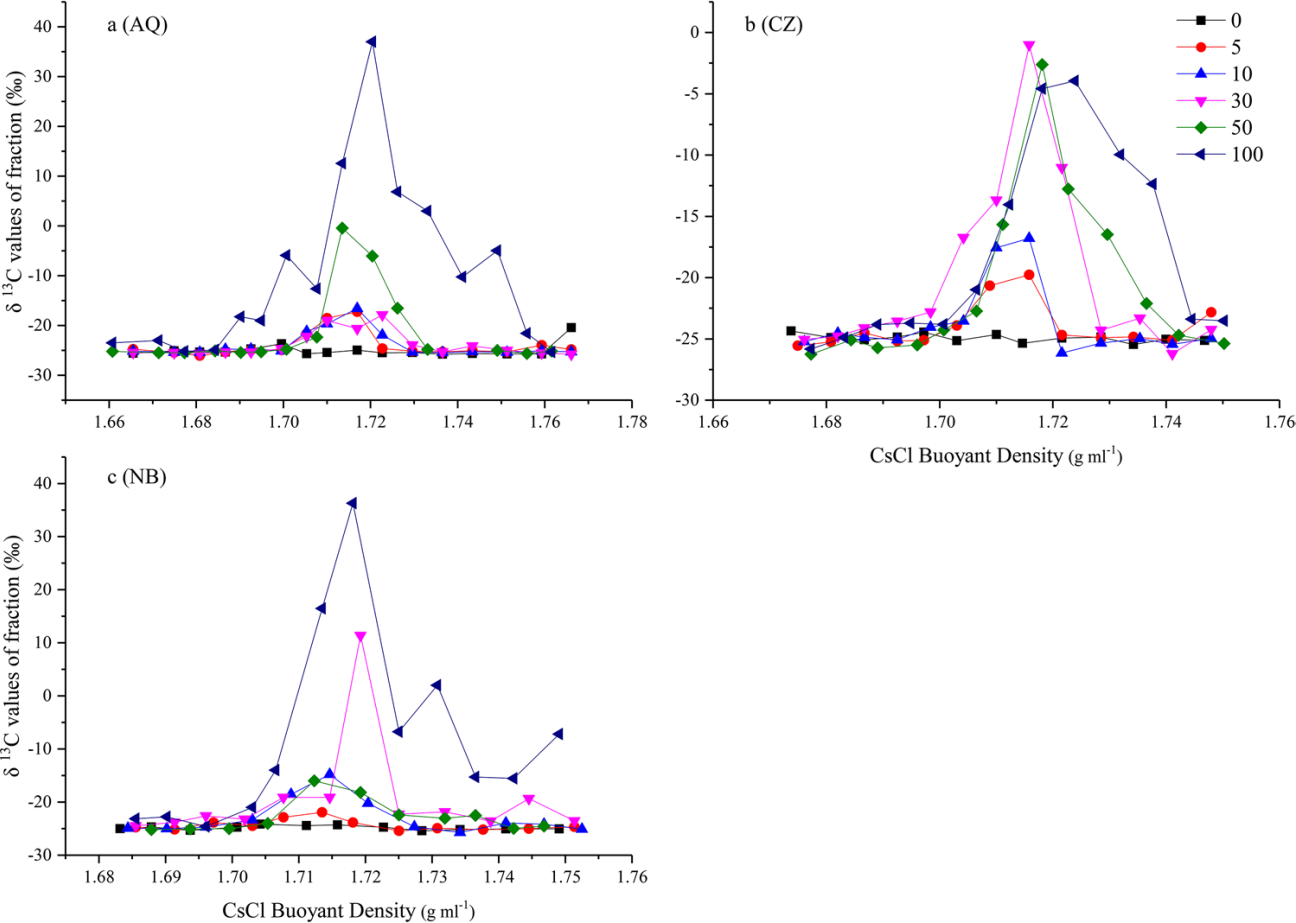
The δ.^13^C values of the DNA from the 3rd to 13th fractions (from heavy to light density) after ultracentrifugation in AQ (**a**), CZ (**b**), and NB (**c**) soils. Bars represent the standard deviation of the mean (*n* = 3)

The δ^13^C values of the fractions in which the 16S or 18S rRNA gene ratio was lower than 0.01 (Figs. 2 and 3) could still be detected and were near to − 25‰ (Fig. 5) and consistent with the δ^13^C value of PEG-6000, which was used for DNA purification after ultracentrifugation. Moreover, certain fractions exhibited labelling even when treated with only 5% ^13^C-glucose in the AQ and CZ soils. The labelled DNA was concentrated in the third to tenth fractions (from heavy to low density) of AQ and NB (Fig. 5a, c) and the fifth to tenth fractions of the CZ soil with pure ^13^C-glucose addition (Fig. 5b). The corresponding labelled 16S rRNA gene was evident in the third to eighth fractions of AQ (Fig. 2a) and the fourth to ninth fractions of the CZ and NB soils (Fig. 2b, c). The corresponding 18S rRNA gene was evident in the fourth to eighth fractions of AQ (Fig. 3a) and in the fifth to tenth fractions of the CZ and NB soils (Fig. 3b, c). In summary, the labelled fractions identified by IRMS included those fractions identified by qPCR.

To precisely determine the labelling, the correlation between the gene abundance difference, obtained as the labelled ratio minus the unlabelled ratio, and the δ^13^C value of the fractionated DNA was analyzed (Table 2). The results revealed a significant correlation in the treatments with pure ^13^C-glucose in all three soils. Considering that the δ^13^C values were similar while the curves did not completely coincide for the unlabelled fractions, the correlation for only the heavy fractions (where the buoyant density was higher than that of the fraction with the maximal gene abundance) was also analyzed (Table 3). As a consequence, treatments with a low ^13^C-glucose content as well as a high ^13^C abundance showed a significant correlation except in the NB and CZ soil at 5% ^13^C-glucose.

**Table 2:** Correlation coefficient between the δ^13^C values of the DNA in all fractions and the difference values of gene abundance ratios from ^13^C-labelled treatments subtracted from the unlabelled control

Soil	Percent of amended ^13^C-glucose	5	10	30	50	100
AQ	16S rRNA gene	0.343	0.514*	0.647**	0.728**	0.634**
	18S rRNA gene	0.270	0.374	0.758**	0.732**	0.574*
CZ	16S rRNA gene	− 0.446	0.520*	0.766**	0.783**	0.777**
	18S rRNA gene	− 0.086	0.283	− 0.083	0.662*	0.722**
NB	16S rRNA gene	0.209	0.399	0.398	0.155	0.601*
	18S rRNA gene	− 0.238	0.210	− 0.093	0.331	0.679*

^*^The mean difference is significant at the 0.05 level

^**^The mean difference is highly significant at the 0.01 level

**Table 3:** Correlation coefficient between the δ^13^C values of the DNA in heavy fractions and the difference values of gene abundance ratios from ^13^C-labelled treatments subtracted from the unlabelled control

Soil	Percent of amended ^13^C-glucose	5	10	30	50	100
AQ	16S rRNA gene	0.975**	0.997**	0.948**	0.969**	0.749*
	18S rRNA gene	0.939**	0.997*	0.921**	0.984*	0.638*
CZ	16S rRNA gene	− 0.206	0.968**	0.984*	0.971**	0.877**
	18S rRNA gene	− 0.713	0.810**	0.995**	0.991**	0.974**
NB	16S rRNA gene	0.950**	0.672*	0.978**	0.933**	0.957**
	18S rRNA gene	0.530	0.716*	0.920**	0.991**	0.970**

The heavy fractions were fractions from that with the maximal gene abundance to that with the heaviest buoyant density

^*^The mean difference is significant at the 0.05 level

^**^The mean difference is highly significant at the 0.01 level

## Discussion

A few studies have detected the δ^13^C values of microbial DNA before executing DNA-SIP. Mao et al. (2014) labelled switchgrass with ^13^CO_2_ for 8 days, and the δ^13^C values of the rhizosphere DNA reached almost 15‰, which approached the values of the AQ soil amended with 10% ^13^C-glucose and the NB soil with 30% ^13^C-glucose in this study. In this experiment, most of the 16S and 18S rRNA genes were still not labelled in the soil, even when amended with 1000 mg/kg pure ^13^C-glucose during a 7-day incubation. However, most bacterial 16S rRNA genes and fungal ITS genes in soil were suitably labelled after being amended with 500 mg/kg pure ^13^C-glucose during 48-h incubation in the studies of Kong et al. (2018). Since the highest ^13^C atom% of the microbial DNA in our study was 1.72%, which occurred in the AQ soil, it is assumed that higher ^13^C-DNA concentrations may have occurred in these earlier studies, which suggests that a higher ^13^C-labelled extent is needed for distinguishing those fractions with the maximal gene abundance between ^13^ and ^12^C treatments after ultracentrifugation in future research. In addition to the difference in the microbial community structure, the magnitude of the background biomass and soil organic carbon content were also important in successful labelling. If the biomass and organic material contents in soil are low, then a relatively large ^13^C biomass will be observed following glucose addition; however, if the background biomass (^12^C) is already large, then the result will always be a lower ratio. This trend may be the reason for the difference between this study and that by Kong et al. (2018), as well as the difference between the NB soil and AQ and CZ soils, because a higher organic carbon content and C/N ratio and smaller ^13^C-labelled DNA amount occurred in the NB soil (Table 1, Fig. 1).

In all three soils treated with 5% ^13^C-glucose, with more than 50 mg/kg ^13^C-glucose, labelling of 16S and 18S rRNA genes was not detected by qPCR. Theoretically, glucose is very easily assimilated by microbes as a carbon and energy source. We assumed that it would be difficult to determine the fraction with ^13^C 16S and 18S rRNA gene fragments when other less readily available carbon sources are added to the soil. In practice, ^13^C-labelled inorganic carbon (CO_2_) or certain organic substrates in addition to glucose have also been added to soil, and DNA-SIP has been conducted in many studies (Beulig et al. 2015; Chen et al. 2018; Li et al. 2018; Zhang et al. 2015). Except for the studies of Beulig et al. (2015), in which labelled and unlabelled DNA were separated based on the quantified DNA concentration with a spectrophotometer after labelling mofette soils with ^13^CO_2_ for 14 days, many other reports indicated difficulties in identifying labelling fractions or directly proceeded with high-throughput sequencing (Da Costa et al. 2018; Li et al. 2018; Zhang et al. 2015). Additionally, a carrier-SIP approach relying on examining the distribution of DNA from ^13^C-labelled and control pure cultures at a CsCl gradient for estimating the ^13^C-labelled DNA of environmental samples was employed in some studies (Liu et al. 2016; Sietio et al. 2018; Sun et al. 2018). Clearly, the carrier-SIP approach neglected the difference in GC content of DNA between the pure culture and environmental samples (Youngblut and Buckley 2014). Overall, precisely estimating the ^13^C-DNA fraction is difficult when the labelling material is not the optimal carbon source, the labelling amount is not sufficient, or the appropriate evaluation methods have not been adopted.

The labelled ratio of DNA is a crucial factor for any DNA-SIP experiment because it affects the extent of DNA divergence in buoyant density after ultracentrifugation. Completely replacing the DNA ^12^C value with ^13^C will increase the buoyant density by approximately 0.04 g cm^−1^ (Birnie 1978; Lueders et al. 2004). The buoyant density of partial 16S and 18S rRNA genes increased from approximately 0.02 to 0.036 and from 0.032 to 0.041, respectively, in the three soils with pure ^13^C-glucose, suggesting that fungi assimilated more glucose than bacteria. Moreover, the increased extent was consistent with previous reports (Birnie 1978; Lueders et al. 2004), although in this study, only a small amount of 16S and 18S rRNA genes was labelled with glucose.

Although qPCR of 16S and 18S rRNA genes was performed before determining the DNA δ^13^C value in each fraction, assessing labelled fractions according to the gene abundance was undoubtedly influenced by the δ^13^C value. Therefore, a gene that had been shifted to a high buoyant density in only one fraction (approximately 0.006 g mL^−1^) was still regarded as labelled by ^13^C in this experiment, while 16 fractions were collected after ultracentrifugation. Otherwise, a shift in two fractions (0.012 g mL^−1^) was required to ensure a reliable labelling result (Orsi et al. 2016). To distinguish the labelled fraction when the qPCR results were inconclusive, the δ^13^C value of each fraction was required.

As in previous studies (Haichar et al. 2008; Haichar et al. 2012), the curve for the δ^13^C values of nucleic acid did not overlap with the amount of nucleic acid in all fractions. It is clear that the maximum δ^13^C values appeared in those fractions with heavier buoyant density than the maximum amount of nucleic acid. Moreover, 2 ~ 4 fractions exhibited notably higher δ^13^C values among the treatments with 5% ^13^C-glucose compared to the control samples (Fig. 5). A significant correlation between the δ^13^C value and the qPCR result was identified for most of the treatments (Table 3). These results suggest that identifying the labelled fractions of 16S and 18S rRNA genes through measuring the δ^13^C values of fractions may be more accurate and can be better visualized than qPCR, especially when the amount of the labelling substrate or the labelling time is not appropriate or the labelling materials are hardly assimilated by microbes, the resultant labelling DNA is insufficient for visualization by qPCR. However, one shortcoming is that the δ^13^C value is the total carbon content in one fraction, while the objective gene is just a part of the DNA in this fraction. Clearly, the method of detecting the δ^13^C values of fractions after ultracentrifugation is best used when bacterial 16S rRNA or fungal ITS/18S rRNA genes are analyzed rather than functional genes. The other shortcoming is that the δ^13^C value will be affected by PEG 6000, which becomes interfused in the DNA solution during the purification process after ultracentrifugation. Because EA-IRMS could detect only the δ^13^C value of the total C in tin, it can underestimate the DNA δ^13^C value and subsequently the number of labelling fractions. Therefore, obtaining a DNA solution that is as pure as possible after ultracentrifugation is essential when measuring the DNA δ^13^C value.

Combining the qPCR and δ^13^C results in this experiment, we inferred that the labelled bacterial 16S rRNA or fungal ITS/18S rRNA genes were mainly distributed in the fractions where the buoyant density was heavier than that of the fraction with the maximum gene abundance. That is, if a fraction has a buoyant density heavier than that of the fraction with the maximum gene abundance and a gene abundance even slightly higher than that of the fraction with the same buoyant density in the unlabelled treatment, the gene in this fraction can be affirmed to be labelled by ^13^C. This finding is also consistent with the results of many studies (Bao et al. 2019; Chen et al. 2018; Sathyamoorthy et al. 2018; Xu et al. 2019). Moreover, determining the gene abundance or δ^13^C values of DNA was only an approach to find the labelling fractions; the specific labelled gene fragments must then be identified by various sequencing technologies and compared between the labelled and unlabeled treatments, or the heavy and light fractions through statistic analysis. Considering that not only one fraction was labelled, sometimes all labelled fractions were sequenced independently or combined to a new subfraction, to avoid losing any of the labelled fragments (Beulig et al. 2015; Pepe-Ranney et al. 2016).

Overall, the separation between the labelled and unlabelled treatments was clearly affected by the DNA ^13^C amount. Generally, the labelled bacterial 16S rRNA and fungal 18S rRNA genes were not present in those fractions with the maximal abundance but in the fractions with a heavier buoyant density. At the same time, fractions with a buoyant density heavier than that of the fractions with the maximum abundance and a higher gene abundance than that of unlabelled corresponding samples could be inferred as being labelled. Measuring the δ^13^C value of the total DNA before ultracentrifugation and that of the fractionated DNA was beneficial in estimating the separation result. However, the DNA solution should be sufficiently purified before δ^13^C determination.

## Data Availability

All datasets and material generated or analyzed in this study are available from the corresponding author upon reasonable request.
